# Firework-related injuries treated at emergency departments in the United States during the COVID-19 pandemic in 2020 compared to 2018–2019

**DOI:** 10.1186/s40621-021-00358-2

**Published:** 2021-11-10

**Authors:** Nathan Maassel, Abbie Saccary, Daniel Solomon, David Stitelman, Yunshan Xu, Fangyong Li, Emily Christison-Lagay, James Dodington

**Affiliations:** 1 Department of Surgery, Yale University School of Medicine, New Haven, CT USA; 2 Department of Emergency Medicine, Yale University School of Medicine, New Haven, CT USA; 3 Department of Surgery, Division of Pediatric Surgery, Yale University School of Medicine, New Haven, CT USA; 4 Yale Center for Analytical Sciences, Yale University School of Public Health, New Haven, CT USA; 5 Department of Pediatrics and Emergency Medicine, Yale University School of Medicine, New Haven, CT USA

## Abstract

**Background:**

Despite a national decrease in emergency department visits in the United States during the first 10 months of the pandemic, preliminary Consumer Product Safety Commission data indicate increased firework-related injuries. We hypothesized an increase in firework-related injuries during 2020 compared to years prior related to a corresponding increase in consumer firework sales.

**Methods:**

The National Electronic Injury Surveillance System (NEISS) was queried from 2018 to 2020 for cases with product codes 1313 (firework injury) and narratives containing “fireworks”. Population-based national estimates were calculated using US Census data, then compared across the three years of study inclusion. Patient demographic and available injury information was also tracked and compared across the three years. Firework sales data obtained from the American Pyrotechnics Association were determined for the same time period to examine trends in consumption.

**Results:**

There were 935 firework-related injuries reported to the NEISS from 2018 to 2020, 47% of which occurred during 2020. National estimates for monthly injuries per million were 1.6 times greater in 2020 compared to 2019 (*p* < 0.0001) with no difference between 2018 and 2019 (*p* = 0.38). The same results were found when the month of July was excluded. Firework consumption in 2020 was 1.5 times greater than 2019 or 2018, with a 55% increase in consumer fireworks and 22% decrease in professional fireworks sales.

**Conclusions:**

Firework-related injures saw a substantial increase in 2020 compared to the two years prior, corroborated by a proportional increase in consumer firework sales. Increased incidence of firework-related injuries was detected even with the exclusion of the month of July, suggesting that the COVID-19 pandemic may have impacted firework epidemiology more broadly than US Independence Day celebrations.

## Background

Firework displays remain an integral part of the American cultural experience, punctuating national and local holiday celebrations, sporting events, fairs, and festivals. Both commercial (Walger et al. 2020) and consumer based firework sales peak in January and July, during New Year’s and Independence Day celebrations, with a corresponding rise in firework-related injuries during these months (Canner et al. 2014). Despite public education, improved firework safety, and down trending annual rates of firework injuries, the distribution of injuries by age and sex has remained largely unchanged since the 1980’s (Billock et al. 2017). Children account for more than 50% of all firework injuries in the US (D'Ippolito et al. 2010), with males being three times more likely to be injured than females (Billock et al. 2017).

The COVID-19 pandemic has had a substantial impact on healthcare utilization in the United States, particularly regarding emergency department (ED) visits. The Centers for Disease Control and Prevention (CDC) reported a 42% decrease in ED visits immediately following declaration of a national emergency for COVID-19 in mid-March, with levels consistently lower than parallel pre-pandemic months until 2021 (Adjemian et al. 2021). Despite this, the Consumer Product Safety Commission (CPSC) preliminarily reported significant increases in emergency department visits related to a number of products including skateboards, scooters, all-terrain vehicles, and fireworks (Schroeder 2021). Additionally, press reports from the American Pyrotechnics Association (APA) suggested an “all time high” for consumer firework purchases during the summer of 2020 (Association 2020). With the pandemic came a series of mandates and prohibitions pertaining to large in-person gatherings and social distancing recommendations. The social changes imparted on society by the pandemic likely influenced the proportion of consumer firework sales during 2020 and therefore may have also influenced the epidemiology of firework injuries during the same time period. We sought to determine rates of firework-related injuries during 2020 compared to years prior, evaluated in conjunction with firework sales data, to direct injury prevention strategies in light of an ongoing global pandemic.

## Methods

**Fig. 1:**
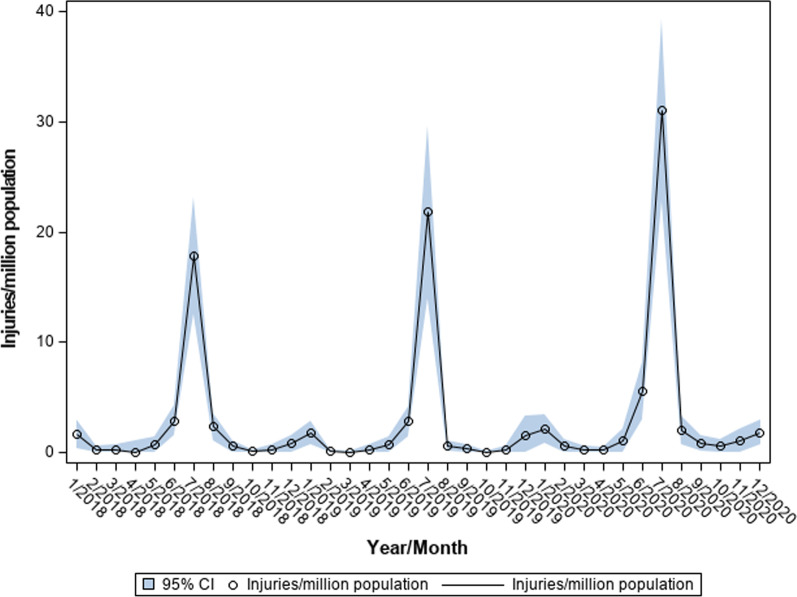
Trends in monthly firework-related emergency department visits in the United States

The National Electronic Injury Surveillance System (NEISS) was used to analyze firework-related injuries. The NEISS database collects information pertaining to consumer product-related injuries from approximately 100 emergency departments across the country under the United States Consumer Product Safety Commission (CPSC). The CPSC then uses these data to generate national estimates for product-related injuries. NEISS data consist of demographic, injury-related, and narrative descriptions for each case (Commission 2020).The NEISS was queried from 2018 to 2020 for all encounters with product codes for firework-related injuries (product code 1313). Additionally, narratives were individually evaluated to confirm injuries related to fireworks. Population-based estimates and 95% confidence intervals of ED visits for firework-related injuries were calculated using US census data and NEISS calculated sample weights. Published estimates from the US Census Bureau were used to calculate population-based estimates for 2020, using the “high” estimate to prevent over-estimation of injuries for that year. Available patient demographics and injury-related information were acquired for each year. The variable specifying body part injury was split into the four most commonly injured individual body parts (hand, eye, finger, face), while all remaining body parts were included in other (lower extremity, trunk, shoulder, foot). Variables such as geographic location of injury and disposition were categorized according to the NEISS data set. National estimates for monthly firework-related injuries per million were compared between 2018, 2019, and 2020 in a time-series analysis using Poisson regression with each month as a cluster to account for within-month correlation of data (Fig. 1). Offset using log transformed population estimate was included in the model to derive incidence rate. Comparisons were performed with and without inclusion of the month July to investigate effects of the pandemic beyond the month with the greatest incidence of injuries.

Additionally, firework sales data were acquired from the American Pyrotechnics Association (APA) for the same time period (2018–2020) to correlate with ED admission data. Firework sales are expressed as consumption in pounds (lbs.) of fireworks and divided into professional (display fireworks) and consumer usage.

Statistical analysis was completed using SAS 9.4 (Cary, NC) software. Statistical significance was set at *p* < 0.05, two-sided. Given that this is a cross-sectional study of a large national dataset, it was not feasible to involve patients or the public in study design. This study was considered exempt from review by the institutional review board of the Yale School of Medicine.

## Results

### Patient demographics and injury data

Between 2018 and 2020, there were 935 cases of firework-related injuries reported within the NEISS, of which 440 (47%) were during 2020. The majority of recorded cases were in patients < age 30 (57–61% of all cases each year). The ‘hand’ was the most frequently injured body part (22–25% of all cases each year). Patient disposition after presentation to the ED was similar between each year, with a slight increase in the number of patients transferred and those that left without being seen in 2020. While the geographic location where injuries took place remained overall similar, there was a slight increase in injuries at home and in the street, with a slight decrease in “other public property”. All available patient and injury information are listed in Table 1. Notably, children and young adults remain the highest age groups by percentage through the years studied (Table 1).

**Table 1:** Raw case counts and patient demographics

Characteristics	2018 *N* = 234	2019 *N* = 261	2020 *N* = 440
*Age, years*			
0–10	21 (9%)	44 (17%)	56 (13%)
11–20	72 (31%)	60 (23%)	124 (28%)
21–30	39 (17%)	51 (20%)	90 (20%)
31–40	36 (15%)	36 (14%)	66 (15%)
41–50	23 (10%)	19 (7%)	29 (7%)
> 50	43 (18%)	51 (20%)	75 (17%)
*Sex*			
Male	170 (73%)	181 (69%)	330 (75%)
*Injured body part*			
Hand	52 (22%)	59 (23%)	108 (25%)
Eye	38 (16%)	37 (14%)	72 (16%)
Finger	40 (17%)	43 (16%)	47 (11%)
Face	23 (10%)	24 (9%)	46 (10%)
Other*	81 (35%)	98 (38%)	167 (38%)
*Disposition*			
Discharged	174 (74%)	202 (77%)	324 (74%)
Transferred	9 (4%)	7 (3%)	26 (6%)
Admitted	45 (19%)	46 (18%)	69 (16%)
Observation	3 (1%)	3 (1%)	3 (1%)
Left without being seen	3 (1%)	3 (1%)	17 (4%)
Fatality	0 (0%)	0 (0%)	1 (0%)
*Location of injury*			
Home	71 (30%)	82 (31%)	144 (33%)
Street	7 (3%)	4 (2%)	16 (4%)
Other public property	22 (9%)	22 (8%)	25 (6%)
School	2 (1%)	0	1 (< 1%)
Place of recreation or sports	11 (5%)	8 (3%)	12 (3%)
Not recorded	121 (52%)	145 (56%)	242 (55%)

*Other for injured body part included lower extremity, trunk, shoulder, foot

### Time-series analysis of monthly firework-related injuries

National estimates for firework-related injuries per million are plotted over time in Fig. 1, demonstrating a small annual surge in January as well as a larger spike during the month of July and lesser increases in the fringe months of June and August. On time series analysis, national estimates for monthly injuries per million were 1.6 times greater in 2020 compared to 2019 (3.9 vs 2.5, respectively, *p* < 0.0001) with no difference between 2018 and 2019 (2.3 vs 2.5, *p* = 0.38). When the month of July is excluded from each year, estimated monthly injuries per million were still 1.9 times greater in 2020 compared to 2019 (1.5 vs 0.8, *p* < 0.0001), again with no difference demonstrated between 2018 and 2019 (0.9 vs 0.8, *p* = 0.49). National estimates for firework-related injuries used to generate Fig. 1 and monthly raw case counts for each year are presented in Table 2.

**Table 2:** Raw case counts and national estimates for monthly firework-related emergency department visits 2018–2020

Month	Raw admission counts			National injury estimates (per million) (95% CI)		
	2018	2019	2020	2018	2019	2020
January	12	19	29	537 (116, 958)	607 (257, 956)	719 (281, 1157)
February	3	4	6	84 (0, 213)	41 (0, 88)	196 (0, 399)
March	3	2	2	104 (0, 256)	20 (0, 53)	72 (0, 189)
April	0	2	4	*	86 (0, 248)	71 (0, 184)
May	5	8	12	242 (0, 486)	240 (26, 455)	368 (10, 726)
June	28	23	52	941 (494, 1389)	926 (470, 1382)	1853 (984, 2722)
July	147	180	283	5823 (4082, 7563)	7150 (4575, 9725)	10,277 (7533, 13,022)
August	19	5	15	766 (361, 1172)	202 (30, 374)	666 (245, 1087)
September	6	3	9	185 (24, 346)	115 (0, 260)	280 (49, 510)
October	2	2	5	35 (0, 83)	25 (0, 66)	191 (0, 417)
November	2	2	7	98 (0, 259)	71 (0, 186)	350 (0, 732)
December	7	11	16	266 (3, 528)	509 (0, 1081)	602 (227, 977)

*Injury estimates in April 2018 could not be calculated due to a lack of recorded ED visits

### Firework sales data

**Fig. 2:**
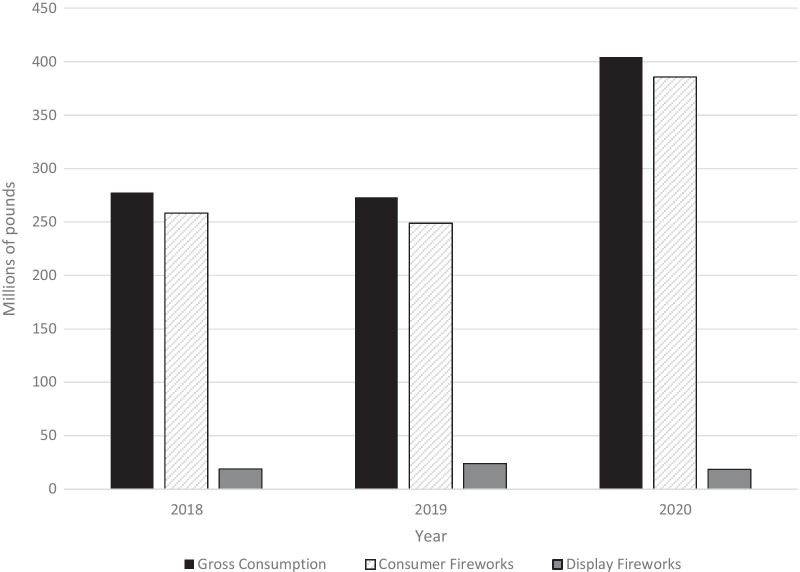
American Pyrotecnics Association data regarding firework consumption in millions of pounds from 2018-2020. In 2020 there was a 1.5 times increase in overall firework consumption and >50% increase in the proportion of consumer firework consumption compared to 2019 and 2018

Between 2018 and 2020, the APA reported 955 million lbs. of fireworks consumed in the US, 42% (401 million lbs.) of which occurred in 2020 (Fig. 2). This is 1.5 times increase over 2019 (273 million lbs.) or 2018 (277.5 million lbs.). Consumer (private) firework consumption increased 55% in 2020 (385.8 million lbs.) compared to 2019 (248.9 million lbs.) and 2018 (258.4 million lbs.). Display (professional) fireworks however saw a 22.41% fall during 2020 (18.7 million lbs.) compared to 2019 (24.1 million lbs.) and 2018 (19.1 million lbs.).

## Discussion

While the COVID-19 pandemic and subsequent quarantines drove down broad categories of ED presentation (with the largest declines observed in the pediatric population), certain types of recreation-driven injuries presented with greater frequency than in pre-pandemic years (Adjemian et al. 2021). This study demonstrates a concerning increase in firework-related injuries in 2020 compared to years prior. An increase in firework-related injuries was observed not only during the “hotspot” month of July, but throughout the year, suggesting that the pandemic impacted the epidemiology of firework-related injuries beyond traditional celebratory clusters. Indeed, the months of June through November saw a 2–6-fold increase in national injury estimates over the same months during the 2 preceding years. This increase in injuries was paralleled by a surge in consumer firework consumption as reported by the APA, with a corresponding drop in professional firework displays in 2020. The startling increase in firework-related injuries highlights the need for constant injury surveillance and most importantly for innovation in injury prevention program development and messaging, based on timely assessment of trends in injury epidemiology. Work by the CPSC in terms of surveillance and dissemination of information, and educational messaging efforts by organizations like the American Academy of Pediatrics should be expanded upon for greater impact.

A national lockdown, reduced public celebrations, and increased time at home may explain the increase in consumer firework sales which led to corresponding rise in injuries. Traditional firework displays produced for large communities were mostly cancelled during 2020 due to social distancing recommendations and federal mandates (Courtemanche et al. 2020; Creswell 2020), yet consumer firework consumption soared. Prior research has correlated increased firework sales with injuries (Morrissey et al. 2021). Furthermore, injuries are more common with consumer fireworks operated by novice operators compared to display fireworks operated by professionals (Canner et al. 2014; Sandvall et al. 2017). Review of the 2020 CPSC annual Fireworks Report revealed that patients aged 0–24 accounted for 49% of all injuries during fiscal year 2020. Fingers, hand, head, face, and eyes are the most commonly injured body parts and could lead to life-long disability (Allison Marier and Lee 2021).

As with all cross-sectional reviews of a national data repository, possible limitations of this study include coding error, misclassification, or underestimation of injury incidence. Furthermore, US public health policy has varied widely by state and by pandemic month resulting in disparate governance over public gatherings and celebrations. Over half of reported data for geographic location is missing in this database; such data might permit a more nuanced understanding of firework usage. Additionally, information regarding case severity is limited, which could impact the significance of the increased incidence of firework-related injury.

This is a large cross-sectional investigation of firework-related injuries presenting to EDs in the US demonstrating a dramatic increase in injuries in 2020 compared to years prior. This is in conjunction with a corresponding rise in firework sales the same year and warrants further investigation into injury severity and strategies for injury prevention.

## Abbreviations

ED: Emergency department
CDC: Centers for Disease Control
CPSC: Consumer Product Safety Commission
APA: American Pyrotechnics Association
NEISS: National Electronic Injury Surveillance System
